# Temporospatial Alterations in Upper-Limb and Mallet Control Underlie Motor Learning in Marimba Performance

**DOI:** 10.3389/fpsyg.2022.834869

**Published:** 2022-02-10

**Authors:** Tristan Loria, Melissa Tan, John de Grosbois, Aiyun Huang, Michael H. Thaut

**Affiliations:** ^1^Music and Health Research Collaboratory (MaHRC), Faculty of Music, University of Toronto, Toronto, ON, Canada; ^2^Rotman Research Institute, Baycrest Health Sciences, Toronto, ON, Canada; ^3^Faculty of Music, University of Toronto, Toronto, ON, Canada

**Keywords:** percussion, motor learning, sensorimotor, motor control, acceleration, upper-limb

## Abstract

Sound-producing movements in percussion performance require a high degree of fine motor control. However, there remains a relatively limited empirical understanding of how performance level abilities develop in percussion performance in general, and marimba performance specifically. To address this issue, nine percussionists performed individualised excerpts on marimba within three testing sessions spaced 29 days apart to assess early, intermediate, and late stages of motor learning. Motor learning was quantified *via* analyses of both the temporal control of mallet movements, and the spatial variability of upper-limb movements. The results showed that temporal control of mallet movements was greater in the intermediate compared to the early learning session, with no significant additional improvements revealed in the late learning session. In addition, spatial variability in the left and right elbows decreased within the intermediate compared to the early learning session. The results suggest that temporal control of mallet movements may be driven by reductions in spatial variability of elbow movements specifically. As a result, this study provides novel evidence for kinematic mechanisms underlying motor learning in percussion which can be applied towards enhancing musical training.

## Introduction

Musicians have a unique challenge when learning the various movements underlying sound-production. Indeed, a significant amount of temporal and spatial coordination of the upper-limbs is required to perform successfully. Percussion performance is a unique musical context because many sound-producing movements share commonalities with non-musical motor tasks (i.e., supination/pronation and flexion/extension of the shoulders, elbows, and wrists). Indeed, these movements are consistent with those executed during activities of daily living such as reaching and grasping. As a result, a percussion practice/performance context can be useful to further understand how complex musical movement patterns develop and change over time, while remaining relevant within the broader sensorimotor control literature. To this end, the present study examined four-mallet marimba performances at three time-points to study motor learning in music.

Instrumental techniques are known to influence sound production in percussion. In a seminal study within this research area, Dahl (2004) investigated drumstick kinematics when performing accented and unaccented notes wherein movements were measured predominantly along the vertical movement axis. The results showed that drummers used wrist and arm movements to adjust stick height and striking velocity when performing stroke techniques such as taps, full-, up-, and down-strokes to yield the desired aural outcome (e.g., playing loud vs. quiet). Further, drummers raised the stick to a greater vertical preparatory height in anticipation of performing an accented relative to unaccented note. Dahl (2004) further reported that when the stick reached its maximum height, the downstroke was created *via* a whiplash-like movement of the wrist. In contrast, unaccented (i.e., soft) strokes were produced with reduced movements of the hand and arm along the vertical axis. Dahl (2004) surmised that limb and drumstick movements were thus intimately linked with sound production (see also Dahl et al., 2011). Nevertheless, sound production in marimba performance can also be influenced by additional factors, such as grip technique.

The grip of the drumstick is a key factor underlying motor control in percussion. A relaxed grip allows for the stick to rebound freely off the drumhead with greater velocity, while a restricted grip facilitates control to stop the stick at a desired rebound height, resulting in a duller sound (e.g., Dahl and Altenmüller, 2008). Performance tempo has also been shown to influence stick and hand movements. At slower tempos, drummers can utilize the time in between strokes to increase their dynamic range, whereas preparatory movements tend to decrease when performing at faster tempi (e.g., Dahl et al., 2011). This work in conjunction with the findings reported above highlight that stick movements and hand position/grip are critical for sound production, thus implicating upper-limb movement patterns as significant contributors to sound production in percussion.

Literature from alternate musical contexts such as piano may further illustrate how upper-limb movements (e.g., elbows, wrists, hands, and fingers) contribute to sound preparation and production. For example, classical pianists organize proximal upper-limb motions in a way that minimizes biomechanical load and muscular effort to the distal muscles (e.g., Furuya and Kinoshita, 2007, 2008). More specifically, organizing movements of the upper-limbs in a proximal-to-distal fashion coincided with increased elbow pronation and supination when performing keystrokes specifically at faster tempi (i.e., Furuya et al., 2011). Expert pianists also optimize the use of gravity during downward arm swings when striking piano keys wherein the effective utilization of the elbow lowered muscular force load at distal limb segments compared to novice performers (i.e., Furuya et al., 2009). Based on the piano kinematics literature cited here, it can be gathered that movements of the elbow may be particularly critical for performance contexts involving melodic instruments like the marimba (cf. non-melodic instruments like the snare drum).

Studies examining the limb kinematics of percussionists at various skill levels have only recently begun to uncover how performance experience refines movement patterns. When performing fast repetitive drumming movements, expert drummers demonstrated greater temporal accuracy which occurred *via* utilizing low-mass distal joints, resulting in whiplash-like movement of the stick towards the drumming surface compared to novices (i.e., Altenmüller et al., 2020; see also Dahl, 2004). Movement smoothness in drummers’ strokes has also been shown to increase at faster tempi while muscular co-articulation linearly decreased in line with slower performance tempo (i.e., Gonzalez-Sanchez et al., 2019, see also Furuya et al., 2012). Further, expert drummers presented with reduced wrist muscle co-contraction resulting in more temporally succinct playing, which was hypothesised to reflect more efficient performance practices (i.e., Beveridge et al., 2020). Naturally, deliberate motor practice alters the capacity to execute movements.

However, studies contrasting expert and trainee musicians provide only a snapshot of movement execution as a function of experience. Critically, such findings cannot account for how movement patterns were acquired and subsequently refined over time. As mentioned above, achieving such understanding has the potential to advance mechanistic knowledge of complex skill execution, as well as advance theories of motor learning in musical contexts. This pursuit may contribute to the development of optimization factors for motor learning in percussion performance which can be used to aid in the training of musicians. To this end, the present study examined motor learning in percussion where upper-limb movements executed within four-mallet marimba performances were assessed from both temporal and spatial perspectives at three time-points [i.e., session (S) 1, S2, and S3]. Briefly, the marimba is a melodic percussion instrument (e.g., a piano with wooden bars) that is played using mallets. By utilizing a marimba approach in the present study, novel insight regarding the development of sound preparation and sound-producing movements occurring along performance axes not previously investigated in alternate percussion investigations (i.e., anteroposterior, and mediolateral) can be examined to further advance pedagogical applications for motor learning in music.

To meet these goals, two main hypotheses were developed and tested. From a temporal perspective, analyses rooted in the frequency domain were utilised to quantify how movements of the mallets changed over time. More specifically, the first (i.e., velocity) and second (acceleration) derivatives of mallet position data were computed. The time-series data for each mallet during each session (i.e., S1, S2, and S3) were converted into a frequency-domain representation using the pwelch method (i.e., relative peak power). Relative peak power reflects the relative amplitude of a particular rate of motion in the acceleration profile and has been utilised to study sensorimotor control of reaching movements (i.e., de Grosbois and Tremblay, 2016, 2018; de Grosbois et al., 2019). In this frequency domain representation, greater peak power values are indicative of high temporal control. As a result, it was hypothesised that relative peak power would increase linearly across the learning sessions (i.e., S1 vs. S2 vs. S3). To assess motor learning from a spatial perspective, variability in limb movements (i.e., hands, wrists, elbows, and shoulders) were computed and compared across testing sessions. In general, reductions in spatial variability of upper-limb movements are considered favourable relative to increases in spatial variability in musical contexts (e.g., Furuya et al., 2009, 2011). It was thus hypothesised that spatial variability of upper-limb movements would reduce across sessions concomitant with the predicted increases in relative peak power. Successfully confirming the hypotheses outlined here would provide novel insight into kinematic factors underlying motor learning in music, which can be leveraged towards pedagogical applications to the training of percussionists.

## Materials and Methods

### Participants

Nine participants (# of females = 4) completed the protocol described below. Participants were recruited from the Percussion Department in the Faculty of Music at the University of Toronto. All participants were pursuing degrees in percussion performance and had an average of 9.3 years (*SD* = 2.1) of percussion experience at the time of participation. All participants self-reported to be right-handed. The study was approved by the University of Toronto Research Ethics Board and participants provided informed written consent prior to the commencement of participation.

### Apparatus

**FIGURE 1:**
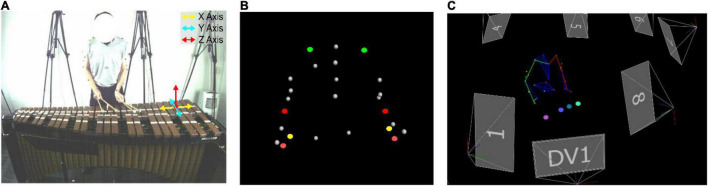
The direction of the movement axes **(A)** are shown. Marker placements (i.e., green = shoulders, red = elbows, yellow = wrists, and salmon = hands) are shown in panel **(B)**. Mallet numbers (i.e., seafoam green = LH1, teal = LH2, purple = RH3, pink = RH4) are shown in panel **(C)**.

Participants performed their excerpts on a Musser Deluxe Studio Grand Rosewood M245 marimba (Ludwig Musser, Elkhart, IN, United States). Motion capture technology was used to gather kinematic data and compute the velocities and acceleration profiles of the markers affixed to the upper-limbs and mallets. The setup included eight Vicon Vero (Vicon Motion Capture, Oxford, United Kingdom) motion capture cameras. Twenty-seven markers were affixed to the limbs of the performers in line with the upper-limb model written in Vicon BodyLanguage (e.g., Murray, 1999). Five markers were positioned on the upper half of the torso including the spinous process of the seventh cervical vertebra, the right scapula, the spinous process of the tenth thoracic vertebra, the jugular notch where the clavicles meet the sternum, and the xiphoid process of the sternum. Markers were further positioned on the left and right limbs including on the acromion-clavicular joints (i.e., used to measure shoulder movements), three inches apart on the upper arms, the lateral epicondyle approximately at the elbow joints (i.e., used to measure elbow movements), the midpoint of the forearms, the thumb side of the radial styloid (i.e., used to measure wrist movements), the little finger side of the ulnar styloids, and just below the third metacarpus on both hands (i.e., used to measure hand movements, see Murray, 1999; Cutti et al., 2005). One marker was positioned at the central point of the ball of each mallet (i.e., four in total). These markers were subsequently used to track movements of each mallet. Given the considerable range of upper-limb and mallet motion during the performance, the markers were sampled at 100 Hz to ensure they could be continuously sampled by the motion capture cameras (see Figure 1).

### Procedure

A total of three testing sessions were completed across a 12-week academic semester at early (i.e., S1), intermediate (i.e., S2), and late (i.e., S3) assessment time-points. All testing sessions were separated by 29-day intervals starting from the first session. This interval was chosen to equate the number of formal lessons between participants. That is, each participant had practice sessions with percussion department faculty members where performance-related feedback and instruction were provided. Separating the testing sessions by 29 days ensured that all participants had an equal number of formal lessons between each assessment time-point. The pieces performed during the testing sessions were part of the individual participant’s curriculum which culminated in an end-of-term evaluation performance. As a result, each participant played an individualised excerpt in each of their early, intermediate, and late testing sessions (i.e., nine excerpts in total). This approach was deemed necessary to study the development and refinement of performance-level motor skills (see section “Discussion”).

There were additional procedural differences between participants. To study performance-level kinematics, participants often performed several trials until they achieved a performance that was representative of their ability to play the piece. More specifically, participants were asked whether they would be satisfied if the performance (i.e., the previous trial) had been given to a concert audience. The minimum number of trials required to achieve the representative trial ranged between 2 and 10 trials, with an average of 3 and a standard deviation (SD) of 2.1. The single representative trial obtained in each session was subject to formal data analysis procedures described below. The length of each trial (i.e., performance) also varied across participants, with an average trial length of 18 s (*SD* = 4 s; range = 14–25 s). Lastly, the tempo of each piece varied between participants with a mean of 87.8 beats per minute (BPM) and a standard deviation of 14.7 (range = 72–115 BPM). Within participants, tempo was always consistent across testing sessions (i.e., each participant performed the piece at the same tempo across sessions). Due to the individualised excerpts and the procedural variability described in this section, data analysis procedures focused on the velocity and acceleration domains to ascertain consistent kinematic patterns across participants.

### Data Analysis

#### Data Reduction—Frequency-Based Mallet Analyses

Acceleration data was computed *via* the positional motion capture data along the three orthogonal cardinal axes (i.e., x, y, and z). Given that the mallets used were unique to each participant, the markers on the mallets were not precisely consistent across participants and sessions. As a result, resultant acceleration was chosen as the primary substrate for the frequency-domain analyses. These resultants were computed as the square-root of the sum of the squared values of each axis, at each time-point. Data from each axis, and the resultant were filtered with a 2nd order lowpass Butterworth filter using a cut-off frequency of 10 Hz. Notably, this filter was applied both before and after the computation of the resultant to minimize the impact of the amplification of noise due to the computation of the resultant acceleration (e.g., Elliott and Hansen, 2010).

The resultant acceleration traces for each participant, mallet, and session were further summarised using frequency-domain analyses. First, a frequency-domain representation of each resultant was computed using the Welch method (i.e., Welch, 1976) as implemented in the SciPy library within Python (Van Rossum and Drake, 1995). The following parameters were implemented when using the Welch method: (1) A 1-second sliding window; (2) A 50% overlap between successive windows; (3) Any linear trend was removed from each window; (4) A Hanning truncation was applied to the window; and (5) Median estimates of power-spectral density were returned. Note that median estimates were returned to minimize the potential influence of artefacts in the signal that may have persisted beyond the signal filtering completed in the first step. Ultimately, this resulted in normalised estimates of the contributions of specific frequencies to the resultant signal. Because participants each played unique pieces, these estimates were further normalised as relative values of the total power to facilitate comparisons across and within participants (see Warner, 1998).

These computed power-spectra-densities were further summarised by extracting two primary measures: (1) The maximum relative amplitude of the largest peak on the spectrum (i.e., relative peak-power); and (2) The width of this peak at 50% of its height (i.e., peak width). The amplitude measure (i.e., relative peak power) represents the relative contribution of the dominant oscillation frequency contributing to the resultant signal. Peak width provides some evidence of the differences in the temporal consistency of the performance, with wider widths indicating varying frequencies contributing to the signal, or at least, increases in temporal variability. Both of these measures were extracted using the find_peaks() function with the Scipy library in Python.

#### Data Reduction—Spatial Variability Analyses

To determine the impact of training on upper-limb movements, spatial variability was computed along the three orthogonal cardinal axes (i.e., x, y, and z). In line with the objective approach for studying temporal performance characteristics in the mallets, spatial characteristics were assessed by obtaining the average standard deviation of individual limb segment movements along each movement axis. Specifically, the standard deviation for movements of the hands, wrists, elbows, and shoulders along each of the x, y, and z axes from the representative trial were computed for each participant. These standard deviation values were then averaged across the nine participants to obtain the mean standard deviation for each movement axis and session (e.g., right elbow variability along the x axis: S1 = 128.3 mm; S2 = 86.8 mm; S3 = 84.1 mm). These values were subject to formal data analysis.

### Statistical Contrasts

The analysis of limb acceleration was completed on both the relative peak power and the peak width estimates obtained *via* the frequency domain analysis, which was completed on the resultant accelerations of the individual mallets. Both dependent variables were examined using a 3 Session (S1 vs. S2 vs. S3) × 2 Hand (Left vs. Right) × 2 Mallet (Thumb vs. Pinky) repeated measures ANOVA. *Post-hoc* analyses of main effects or interactions involving the continuous variable of Session were completed using single degree-of-freedom polynomial contrasts. Otherwise, *post-hoc* pairwise comparisons were completed, and a Bonferroni correction was applied, resulting in an adjusted alpha threshold of *p* = 0.02. If a violation of sphericity was observed, The Greenhouse-Geisser correction was applied to the degrees of freedom before evaluating statistical significance.

The analysis of spatial variability was completed for the standard deviation of movements from all limb segments under study (i.e., hands, wrists, elbows, and shoulders). Standard deviation values were submitted to separate repeated measures ANOVAs for each limb segment and movement axis with session as the within subjects factor (i.e., S1 vs. S2 vs. S3). Only the limb segments that resulted in a significant effect of session were reported below. Main effects of session were followed up with Bonferroni corrected *t-*tests with a corrected alpha threshold of *p* = 0.02 as *post-hoc* procedures.

## Results

### Frequency-Based Mallet Analyses

**FIGURE 2:**
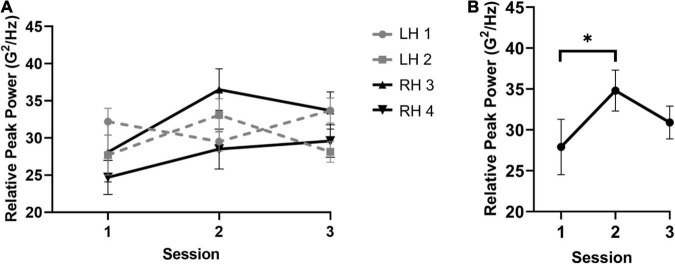
Results for the frequency-based analysis of mallet movements. The overall pattern of effects for peak power and the significant mallet × session interaction is shown in panels **(A,B)**, respectively. The analysis revealed that relative peak power increased specifically for the thumb mallets between sessions 1 and 2. *indicates significance at the Bonferroni corrected alpha threshold.

The acceleration analysis for relative peak power failed to reveal a main effect of session, hand, or mallet (i.e., all *p* values > 0.06). However, a significant interaction between session and mallets was observed, *F*(1.4,11.2) = 6.8, *p* = 0.017, $\eta_{p}^{2}$ = 0.39 (see Figure 2). Follow-up simple effects analyses showed a significant modulation only for mallets 2 and 3 (i.e., thumb) across sessions, *F*(2,16) = 5.5, *p* = 0.02, $\eta_{p}^{2}$ = 0.41. The resulting polynomial contrast conducted across sessions and collapsed between mallets 2 and 3 showed a significant negative quadratic trend across sessions, *F*(1,16) = 8.83, *p* = 0.009, $\eta_{p}^{2}$ = 0.36. Examination of the quadratic trend indicated that the greatest increase in relative peak power occurred for most participants in S2 (*M* = 34.8 G^2^/Hz, *SD* = 6.4) relative to S1 (*M* = 27.9 G^2^/Hz, *SD* = 9.3). These results likely indicate that the relative contribution of the dominant rate of acceleration increases specifically between S1 and S2. In other words, performance variability at the peak frequency contributed to the performance overall to a greater extent during S2. No significant effects were observed for the peak width. In the absence of significant scaling of the peak width, the increase in relative peak power likely reflects a reduction on task-irrelevant temporal variability in the thumb mallets specifically. Thus, participants may be constraining movement degrees of freedom within the intermediate stages of learning to focus on executing unique mallet movements in line with temporal accuracy constraints.

### Spatial Variability Analyses

**FIGURE 3:**
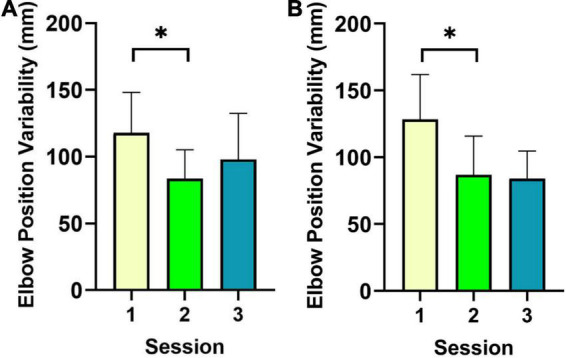
Results for spatial variability of elbow movements are shown. Results for both elbows [i.e., left elbow **(A)**, right elbow **(B)**] revealed reduced spatial variability between S1 and S2. *indicates significance at the Bonferroni corrected alpha threshold.

The only significant effects of Session were observed in the elbows. Indeed, spatial variability of elbow movements yielded significant effects of Session for both the left, *F*(2,16) = 4.8, *p* = 0.02, $\eta_{p}^{2}$ = 0.38, and right, *F*(2,16) = 6.5, *p* = 0.009, $\eta_{p}^{2}$ = 0.45 elbows along the mediolateral x axis. For left elbow position variability, *post-hoc* contrasts revealed greater variability in S1 (*M* = 117.7 mm, *SD* = 30.5) compared to S2 (*M* = 83.6 mm, *SD* = 21.7), *t*(8) = 3.1, *p* = 0.01, 95% CI = [8.9, 59.4]. In the right elbow, position variability was greater in S1 (*M* = 128 mm, *SD* = 33.5) compared to S2 (*M* = 86.8 mm, *SD* = 29), *t*(8) = 3.4, *p* = 0.01, 95% CI = [13.2, 69.8]. Results for both elbows are shown in Figure 3. This pattern of effects may indicate that improved spatial variability in elbow movements may be linked to the reduced temporal variability in the mallets. This hypothesis was further considered below.

## Discussion

This study investigated how upper-limb movement patterns in a percussion context are acquired and refined from spatial and temporal perspectives at three time-points representing early, intermediate, and late phases of motor learning. The results indicated that high spatial and temporal variability in limb movements and mallet acceleration within early learning stages was subsequently reduced in intermediate stages. The strongest effects for temporal variability were observed between S1 and S2, which was unexpected due to the anticipated linear nature of improvement hypothesised above. Instead, the kinematic changes observed specifically within S2 suggest that a reduction on task-irrelevant temporal variability occurs following only one month of training, and predominantly occurs for the inside mallets (i.e., thumb mallets 2 and 3). Spatial alterations in elbow variability likely contributed to these temporal effects. That is, reductions in spatial variability followed a similar trajectory as the temporal effects observed in the mallets. Such findings provide critical insight into the development of sensorimotor control underlying motor learning in music.

As previously mentioned, temporal data obtained from mallet acceleration revealed that the most consistent change in relative peak power overall was between S1 and S2. Relative peak power was used to quantify movements of the individual mallets across the three testing sessions, wherein increases in relative peak power indicated a greater isolation of the predominant temporal rate of performance (e.g., de Grosbois and Tremblay, 2016, 2018). A unique finding of the present study was that changes in relative peak power were primarily driven by movement alterations occurring at the thumb mallets (i.e., mallets 2 and 3), which was not observed in the outer mallets (mallets 1 and 4). This effect may provide novel insight into the time course of motor learning in four-mallet marimba performance. That is, the control of inner vs. outer mallets follow unique learning trajectories, with motor control of the inner mallets developing more rapidly compared to the outer mallets. Such a hypothesis may be supported by differences in visual feedback of outer and inner mallet trajectories.

Asymmetries are a common constraint in motor behaviour. Indeed, it is well known that manual asymmetries exist within the upper-limbs, with humans often showing clear hand dominance (e.g., Oldfield, 1971; Sainburg and Kalakanis, 2000). Asymmetries can further be found within the visual system wherein visual feedback of the moving limb gathered by the dominant eye may be critical for the online control of pointing movements (i.e., Manzone et al., 2018; Loria et al., 2019). The learning asymmetries between the outer and inner mallets were consistent with the asymmetries literature, and suggest the presence of dominant (i.e., inner) vs. non-dominant (i.e., outer) mallets. Such an effect may arise because the outer mallets are often in the peripheral visual field during the performance, thus limiting the quality of visual feedback required to refine trajectories over time (e.g., Sivak and MacKenzie, 1990; Proteau et al., 2000; Loftus et al., 2004; King et al., 2010). As a direction of future pursuit, alternative interventions that manipulate the availability of visual feedback in peripheral and central vision in four-mallet marimba performance can further evaluate visuomotor contributions to learning.

The results also indicated that the greatest reduction in spatial variability occurred between S1 and S2 in both elbows along the mediolateral movement axis. Reduced spatial variability of elbow movements has previously been shown to delineate experienced vs. inexperienced drummers, with the former showing lower variability overall (e.g., Altenmüller et al., 2020). The novel contribution of this investigation is that reductions in elbow spatial variability coincided with increases in the relative peak power (i.e., the predominant performance frequency) of mallet movements. As a result, it may be postulated that decreased spatial variability across sessions likely served to decrease task-irrelevant variability at the level of the mallet. This pattern may suggest that a decrease in elbow movement variability results in greater control of distal thumb mallet movements specifically. Support for this hypothesis may be found in cello performance, wherein precise spatial control of right elbow and shoulder movements was associated with lower bow movement variability underlying sound production (i.e., Verrel et al., 2014; Gonzalez-Sanchez et al., 2019). As a result, the coupling of spatial and temporal control may be one mechanism underlying motor learning in marimba specifically and music performance in general.

The results also have implications for the development of movements not directly related to sound production. Contrary to the stated hypotheses, relative peak power did not increase linearly during training but appeared to plateau between S2 and S3 for the thumb mallets (see Figure 2). This pattern likely reflects changes in practice foci during the motor skill acquisition process. Following S2, participants likely reached optimal sensorimotor and kinematic stability related to the movements underlying sound production (i.e., effective gestures), and thus emphasised the development of expressive gestures. Previous work has clearly demonstrated the vital role of expressive gestures, including how such gestures can be leveraged to influence the perceived aural quality of a performance (e.g., Davidson, 1993; Schutz and Lipscomb, 2007; Broughton and Davidson, 2016; Wanderley and Vines, 2016). Although the present study focused on motor learning of sound-producing movements, the results may be interpreted as evidence that expressive gestures emerge one month into training once sensorimotor learning of sound-producing movements has occurred.

Prior to concluding, it is pertinent to highlight some of the limitations of this study. Most critically, kinematic changes observed during testing cannot be linked directly to specific feedback or approaches to training provided during individual lessons. In addition, this study examined a small sample where only the percussion students at the host institution who were actively practicing marimba repertoire were involved. Lastly, all participants performed unique excerpts. Regarding the potential mechanistic impact of this study, however, it is important to consider that the observed reductions in temporal and spatial variability did occur across different musical pieces, thus highlighting the potential stability of the effects reported here. In addition, all participants were actively preparing their pieces for an end-of-term evaluation performance, which supports the external validity of this study. However, it is recommended that future investigations test the hypotheses proposed here using a larger sample of students from multiple percussion departments and music schools.

Nevertheless, this study still provided valuable insight into the motor skill acquisition process in percussion performance which to date has been limited. This study demonstrated that high temporospatial variability in early learning stages was reduced within intermediate stages when performance motions stabilised. The rate of change between temporal and spatial learning also appeared to be linked. Following one month of training, a relatively permanent change in elbow and dominant thumb mallet movements occurred that was stable over time. Therefore, participants may be constraining elbow movement degrees of freedom within intermediate phases of learning to focus particularly on temporal characteristics and the development of expressive gestures. These findings may also be relevant for other contexts such as sports where elbow movements are critical to task success including overarm throwing in baseball (e.g., Hore et al., 2005; Hore and Watts, 2011). Importantly, this study represents a first step towards understanding mechanisms of motor learning in marimba performance which can be leveraged in future pedagogical approaches to enhance the training of musicians.

## Data Availability Statement

The raw data supporting the conclusions of this article will be made available by the authors, without undue reservation.

## Ethics Statement

The studies involving human participants were reviewed and approved by University of Toronto Research Ethics Board. The patients/participants provided their written informed consent to participate in this study.

## Author Contributions

TL designed the study, collected and analysed the data, and wrote the manuscript. MT analysed the data and wrote the manuscript. JG designed and conducted the frequency-based analysis, and wrote the manuscript. AH secured the funding, helped to recruit participants, and wrote the manuscript. MHT secured the funding and wrote the manuscript. All authors contributed to the article and approved the submitted version.

## Conflict of Interest

The authors declare that the research was conducted in the absence of any commercial or financial relationships that could be construed as a potential conflict of interest.

## Publisher’s Note

All claims expressed in this article are solely those of the authors and do not necessarily represent those of their affiliated organizations, or those of the publisher, the editors and the reviewers. Any product that may be evaluated in this article, or claim that may be made by its manufacturer, is not guaranteed or endorsed by the publisher.
